# DNA from Dust: Comparative Genomics of Large DNA Viruses in Field Surveillance Samples

**DOI:** 10.1128/mSphere.00132-16

**Published:** 2016-10-05

**Authors:** Utsav Pandey, Andrew S. Bell, Daniel W. Renner, David A. Kennedy, Jacob T. Shreve, Chris L. Cairns, Matthew J. Jones, Patricia A. Dunn, Andrew F. Read, Moriah L. Szpara

**Affiliations:** a Department of Biochemistry and Molecular Biology, Center for Infectious Disease Dynamics, and the Huck Institutes of the Life Sciences, Pennsylvania State University, University Park, Pennsylvania, USA; b Center for Infectious Disease Dynamics, Departments of Biology and Entomology, Pennsylvania State University, University Park, Pennsylvania, USA; c Department of Veterinary and Biomedical Sciences, Pennsylvania State University, University Park, Pennsylvania, USA; Northwestern University Feinberg School of Medicine

**Keywords:** Marek’s disease virus, genomics, herpesviruses, polymorphism, virulence

## Abstract

The intensification of the poultry industry over the last 60 years facilitated the evolution of increased virulence and vaccine breaks in Marek’s disease virus (MDV-1). Full-genome sequences are essential for understanding why and how this evolution occurred, but what is known about genome-wide variation in MDV comes from laboratory culture. To rectify this, we developed methods for obtaining high-quality genome sequences directly from field samples without the need for sequence-based enrichment strategies prior to sequencing. We applied this to the first characterization of MDV-1 genomes from the field, without prior culture. These viruses were collected from vaccinated hosts that acquired naturally circulating field strains of MDV-1, in the absence of a disease outbreak. This reflects the current issue afflicting the poultry industry, where virulent field strains continue to circulate despite vaccination and can remain undetected due to the lack of overt disease symptoms. We found that viral genomes from adjacent field sites had high levels of overall DNA identity, and despite strong evidence of purifying selection, had coding variations in proteins associated with virulence and manipulation of host immunity. Our methods empower ecological field surveillance, make it possible to determine the basis of viral virulence and vaccine breaks, and can be used to obtain full genomes from clinical samples of other large DNA viruses, known and unknown.

**IMPORTANCE** Despite both clinical and laboratory data that show increased virulence in field isolates of MDV-1 over the last half century, we do not yet understand the genetic basis of its pathogenicity. Our knowledge of genome-wide variation between strains of this virus comes exclusively from isolates that have been cultured in the laboratory. MDV-1 isolates tend to lose virulence during repeated cycles of replication in the laboratory, raising concerns about the ability of cultured isolates to accurately reflect virus in the field. The ability to directly sequence and compare field isolates of this virus is critical to understanding the genetic basis of rising virulence in the wild. Our approaches remove the prior requirement for cell culture and allow direct measurement of viral genomic variation within and between hosts, over time, and during adaptation to changing conditions.

## Abstract (precis)

Despite both clinical and laboratory data that show increased virulence in field isolates of MDV-1 over the last half century, we do not yet understand the genetic basis of its pathogenicity. Our knowledge of genome-wide variation between strains of this virus comes exclusively from isolates that have been cultured in the laboratory. MDV-1 isolates tend to lose virulence during repeated cycles of replication in the laboratory, raising concerns about the ability of cultured isolates to accurately reflect virus in the field. The ability to directly sequence and compare field isolates of this virus is critical to understanding the genetic basis of rising virulence in the wild. Our approaches remove the prior requirement for cell culture and allow direct measurement of viral genomic variation within and between hosts, over time, and during adaptation to changing conditions.

## INTRODUCTION

Marek’s disease virus (MDV), a large DNA alphaherpesvirus of poultry, became increasingly virulent over the second half of the 20th century, evolving from a virus that caused relatively mild disease to one that can kill unvaccinated hosts lacking maternal antibodies in as little as 10 days (1–5). Today, mass immunizations with live attenuated vaccines help to control production losses, which are mainly associated with immunosuppression and losses due to condemnation of carcasses (4, 6). Almost 9 billion broiler chickens are vaccinated against MD each year in the United States alone (7). MD vaccines prevent host animals from developing disease symptoms, but do not prevent them from becoming infected, nor do they block transmission of the virus (6, 8). Perhaps because of that, those vaccines may have created conditions favoring the evolutionary emergence of the hyperpathogenic strains that dominate the poultry industry today (5). Certainly, virus evolution undermined two generations of MD vaccines (1–4). However, the genetics underlying MDV-1 evolution into more virulent forms and vaccine breaks are not well understood (4, 9). Likewise, the nature of the vaccine break lesions that can result from human immunization with live-attenuated varicella zoster virus (VZV) vaccine is an area of active study (10–13).

Remarkably, our understanding of MDV-1 (genus *Mardivirus*, species *Gallid alphaherpesvirus type 2*) genomics and genetic variation comes exclusively from the study of 10 different laboratory-grown strains (14–21). Most herpesviruses share this limitation, where the large genome size and the need for high-titer samples has led to a preponderance of genome studies on cultured virus, rather than clinical or field samples (22–28). Repeated observations about the loss of virulence during serial passage of MDV-1 and other herpesviruses raise concerns about the ability of cultured strains to accurately reflect the genetic basis of virulence in wild populations of virus (25, 29–31). The ability to capture and sequence viral genomes directly from host infections and sites of transmission is the necessary first step to reveal when and where variations associated with vaccine breaks arise and which ones spread into future host generations, as well as to begin to understand the evolutionary genetics of virulence and vaccine failure.

Recent high-throughput sequencing (HTS) applications have demonstrated that herpesvirus genomes can be captured from human clinical samples using genome amplification techniques such as oligonucleotide enrichment and PCR amplicon-based approaches (32–36). Here we present a method for the enrichment and isolation of viral genomes from dust and feather follicles, without the use of either of these solution-based enrichment methods. Chickens become infected with MDV by the inhalation of dust contaminated with virus shed from the feather follicles of infected birds. Although these vaccinated hosts were infected by and shedding wild MDV-1, there were no overt disease outbreaks. Deep sequencing of viral DNA from dust and feather follicles enabled us to observe, for the first time, the complete genome of MDV-1 directly from field samples of naturally infected hosts. This revealed variations in both new and known candidates for virulence and modulation of host immunity. These variations were detected both within and between the virus populations at different field sites and during sequential sampling. One of the new loci potentially associated with virulence, in the viral transactivator ICP4 (MDV084/MDV100), was tracked using targeted gene surveillance of longitudinal field samples. These findings confirm the genetic flexibility of this large DNA virus in a field setting and demonstrate how a new combination of HTS and targeted Sanger-based surveillance approaches can be combined to understand viral evolution in the field.

## RESULTS

### Sequencing, assembly, and annotation of new MDV-1 consensus genomes from the field.

**FIG 1:**
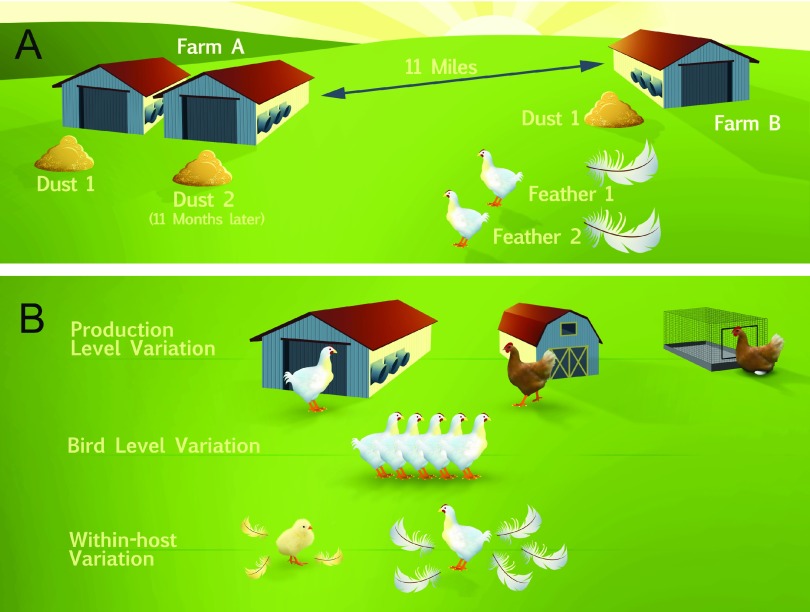
Diagram of samples collected for genome sequencing of field isolates of MDV. (A) Samples collected for genome sequencing were sourced from two Pennsylvania farms with large-scale operations that house approximately 25,000 to 30,000 individuals per building. These farms were separated by 11 mi. On Farm A, two separate collections of dust were made 11 months apart. On Farm B, we collected one dust sample and individual feathers from several hosts, all at a single point in time. In total, three dust collections and two feathers were used to generate five consensus genomes of MDV field isolates (Table 1). (B) These methods can be used to explore additional aspects of variation in future studies. (Images are courtesy of Nick Sloff, Department of Entomology, Penn State University, reproduced with permission.)

To assess the level of genomic diversity within and between field sites that are under real world selection, two commercial farms in central Pennsylvania (11 mi. apart) with a high prevalence of MDV-1 were chosen (Fig. 1A). These operations raise poultry for meat (also known as broilers) and house 25,000 to 30,000 individuals per house. The poultry were vaccinated with a bivalent vaccine composed of MDV-2 (strain SB-1) and herpesvirus of turkeys (HVT [strain FC126]). In contrast to the Rispens vaccine, which is an attenuated MDV-1 strain, MDV-2 and HVT can be readily distinguished from MDV-1 across the length of the genome, which allowed us to differentiate wild MDV-1 from concomitant shedding of vaccine strains. These farms are part of a longitudinal study of MDV-1 epidemiology and evolution in modern agricultural settings (38).

To obtain material for genomic surveillance, we isolated MDV nucleocapsids from dust or epithelial tissues from the individual feather follicles of selected hosts (see Materials and Methods; in the supplemental material, see Fig. S1 and S2 for overviews and Tables S1 and S2 for DNA yields). A total of five uncultured wild-type samples of MDV were sequenced using an in-house Illumina MiSeq sequencer (Table 1, Illumina MiSeq output [see Materials and Methods for details]). The sequence read data derived from dust contained approximately 2 to 5% MDV-1 DNA, while the feather samples ranged from ~27% to 48% MDV-1 (Table 1, percentage of MDV-specific reads). Since dust represents the infectious material that transmits MDV from host to host and across generations of animals that pass through a farm or field site, we pursued analysis of wild MDV-1 genomes from both types of source material.

10.1128/mSphere.00132-16.1 Figure S1  Procedures for enrichment and isolation of MDV DNA from dust or individual feather follicles. (A) Procedure for enrichment of MDV DNA using dust as the source of viral DNA. Vortexing, centrifugation, and sonication were essential to release cell-associated virus into the solution. The virus-containing supernatant was then passed through a 0.8-µm-pore filter, and the viral particles were captured using a 0.1-µm-pore filter. The membrane of the 0.1-µm filter was then excised and used for extraction of the viral DNA. (B) Procedure for enrichment of MDV DNA using chicken feather follicle as the source of viral DNA. A feather was mechanically disrupted (bead beating) and treated with trypsin to break open host cells and release cell-free virus into the solution. The sample was then treated with DNase to remove contaminant DNA. Finally, the viral capsids were lysed to obtain viral genomic DNA. Download Figure S1, PDF file, 1.1 MB. Copyright © 2016 Pandey et al. 2016 Pandey et al. This content is distributed under the terms of the Creative Commons Attribution 4.0 International license.

10.1128/mSphere.00132-16.2 Figure S2  Workflow for computational enrichment for MDV sequences and subsequent viral genome assembly and taxonomic profiling. The VirGA workflow (39) requires an input of high-quality HTS data from the viral genome of interest. For this study, we added an additional step that selected MDV-like sequence reads from the milieu of dust and feather samples. The sequence reads of interest were obtained by using BLAST to compare all reads against a custom MDV database with an E value of 10^−2^; these were then submitted to VirGA for assembly. Taxonomic profiling followed a similar path using NCBI’s all-nucleotide database to identify the taxonomic kingdom for each sequence read. In this workflow diagram, parallelograms represent data outputs, while rectangles represent computational actions. Download Figure S2, PDF file, 0.9 MB. Copyright © 2016 Pandey et al. 2016 Pandey et al. This content is distributed under the terms of the Creative Commons Attribution 4.0 International license.

10.1128/mSphere.00132-16.5 Table S1  Yield and percentage of MDV1 plus MDV2 and total nanograms of DNA in each sample for Farm A-dust 1, Farm A-dust 2, and Farm B-dust. Download Table S1, PDF file, 0.1 MB. Copyright © 2016 Pandey et al. 2016 Pandey et al. This content is distributed under the terms of the Creative Commons Attribution 4.0 International license.

**TABLE 1:** Field sample statistics and assembly of MDV-1 consensus genomes

Sample	Sample preparation			Illumina MiSeq output			New viral genomes		
	ng DNA	% MDV-1	% MDV-2	Total no. of reads^a^	No. of MDV-specific reads^a^	% MDV-specific reads	Avg depth (fold)	Genome length (bp)	NCBI accession no.
Farm A-dust 1	120	2.4	4.6	1.4 × 10^7^	3.7 × 10^5^	2.6	271	177,967	KU173116
Farm A-dust 2	127	1.3	2.7	2.5 × 10^7^	5.1 × 10^5^	2.0	333	178,049	KU173115
Farm B-dust	144	0.6	5.9	2.7 × 10^7^	1.4 × 10^6^	5.2	597	178,169	KU173119
Farm B-feather 1	12	40.6	0.1	3.9 × 10^5^	1.0 × 10^5^	26.9	44	178,327	KU173117
Farm B-feather 2	27	5.7	0	3.4 × 10^5^	1.7 × 10^5^	48.3	68	178,540	KU173118

a The sequence read counts shown are the sum of forward and reverse reads for each sample.

**FIG 2:**
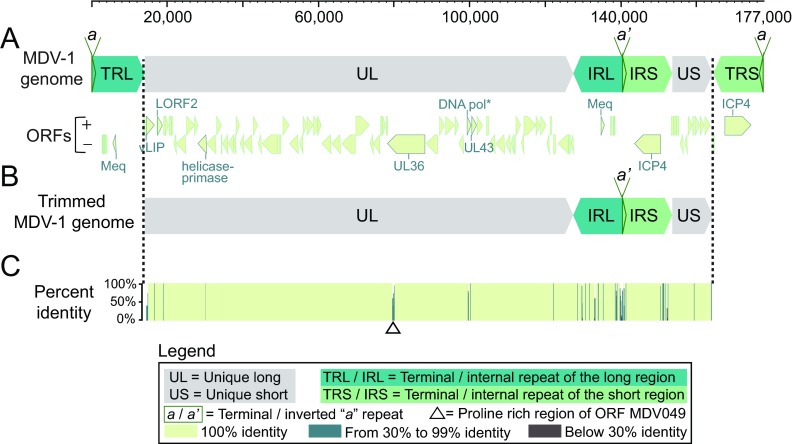
The complete MDV-1 genome includes two unique regions and two sets of large inverted repeats. (A) The full structure of the MDV-1 genome includes a unique long region (UL) and a unique short region (US), each of which is flanked by large repeats known as the terminal and internal repeats of the long region (TRL and IRL) and the short region (TRS and IRS). Most ORFs (pale green arrows) are located in the unique regions of the genome. ORFs implicated in MDV pathogenesis are outlined and labeled: these include ICP4 (MDV084/MDV100), UL36 (MDV049), and Meq (MDV005/MDV076) (see Results for a complete list). (B) A trimmed-genome format without the terminal repeat regions was used for analyses in order to not overrepresent the repeat regions. (C) Percentage of identity from mean pairwise comparison of five consensus genomes, plotted spatially along the length of the genome. Darker colors indicate lower percentages of identity (see Legend).

Consensus genomes were created for each of the five samples in Table 1, using a recently described combination of *de novo* assembly and reference-guided alignment of large sequence blocks, or contigs (Fig. 2A) (39). Nearly complete genomes were obtained for all five samples (Table 1). The coverage depth for each genome was directly proportional to the number of MDV-1-specific reads obtained from each sequencing library (Table 1, MDV-specific reads and average [fold] depth). The dust sample from Farm B had the highest coverage depth, at an average of almost 600× across the viral genome. Feather 1 from Farm B had the lowest coverage depth, averaging 44× genome-wide, which still exceeds that of most bacterial or eukaryotic genome assemblies. The genome length for all 5 samples was approximately 180 kb (Table 1), which is comparable to all previously sequenced MDV-1 isolates (14–21).

**FIG 3:**
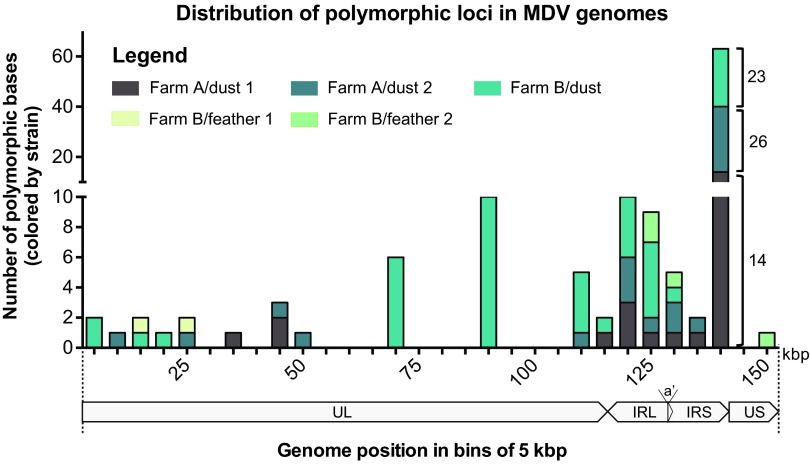
Genome-wide distribution of polymorphic bases within each consensus genome. Polymorphic base calls from each MDV genome were grouped in bins of 5 kb, and the sum of polymorphisms in each bin was plotted. Farm B-dust (aqua) contained the largest number of polymorphic bases, with the majority occurring in the repeat region (IRL/IRS). Farm A-dust 1 (brown) and Farm A-dust 2 (gray) harbored fewer polymorphic bases, with a similar distribution to Farm B-dust. Polymorphic bases detected in feather genomes were more rare, although this likely reflects their lower coverage depth (see Table 1). Note that the upper and lower segments of the *y* axis have different scales; the numbers of polymorphic bases per genome for the split column on the right are labeled for clarity.

**FIG 4:**
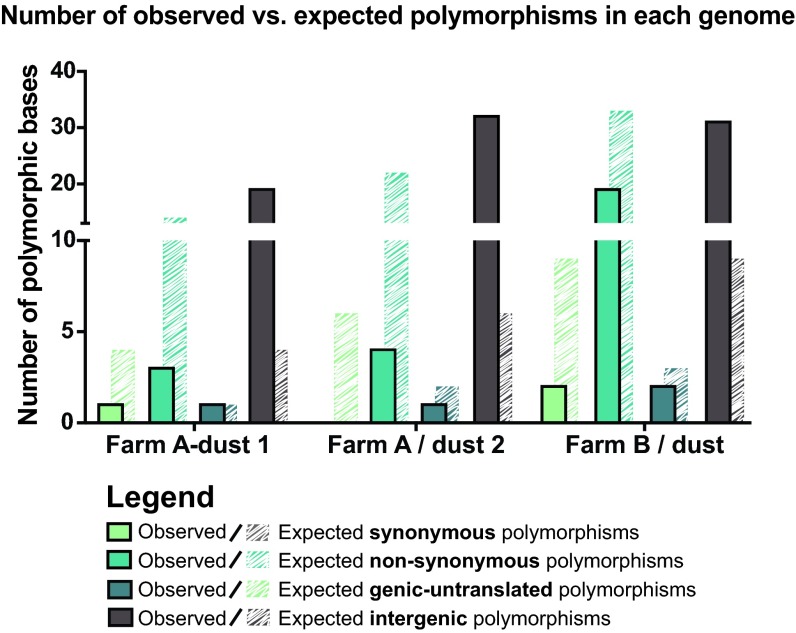
Observed versus expected polymorphism categories for each consensus genome. Each consensus genome was analyzed for the presence of polymorphic loci (see Materials and Methods for details). Observed polymorphic loci (solid bars) were categorized as causing synonymous (green) or nonsynonymous (aqua) mutations or as genic untranslated (gray) or intergenic (brown). The expected outcomes (striped bars) for a random distribution of polymorphisms are plotted behind the observed outcomes (solid bars) for each category. For all genomes, there was a significant difference of the observed versus expected intergenic polymorphisms relative to those of other categories.

For each field sample collected and analyzed here, we assembled a consensus viral genome. We anticipated that the viral DNA present in a single feather follicle might be homotypic, based on similar results found for individual vesicular lesions of the alphaherpesvirus VZV (10, 33). We further expected that the genomes assembled from a dust sample would represent a mix of viral genomes, summed over time and space. Viral genomes assembled from dust represent the most common genome sequence, or alleles therein, from all of the circulating MDV-1 viruses on a particular farm. The comparison of consensus genomes provided a view into the amount of sequence variation between Farm A and Farm B or between two individuals on the same farm (Table 2). In contrast, examination of the polymorphic loci within each consensus genome assembly allowed us to observe the level of variation within the viral population at each point source (Fig. 3 to 4; see Fig. S3 and Table S3 in the supplemental material).

10.1128/mSphere.00132-16.3 Figure S3  Genome-wide distribution of polymorphisms within each consensus genome, using high-stringency criteria. Polymorphic base calls from each MDV genome were grouped by position in bins of 5 kb, and the sum of polymorphisms in each bin was plotted. Stricter parameters of polymorphism detection (see Materials and Methods for details) revealed a similar distribution to those in Fig. 3. No polymorphisms were detected in feather-derived genomes using high-stringency criteria, due to their lower coverage depth (Table 1). Note that the upper and lower segments of the *y* axis have different scales; the numbers of polymorphic bases per segment for the split column on the right are thus labeled on the graph. Download Figure S3, PDF file, 0.9 MB. Copyright © 2016 Pandey et al. 2016 Pandey et al. This content is distributed under the terms of the Creative Commons Attribution 4.0 International license.

10.1128/mSphere.00132-16.6 Table S2  Yield and percentage of MDV1 plus MDV2 and total nanograms of DNA in each sample for Farm B-feather 1 and Farm B-feather 2. Download Table S2, PDF file, 0.1 MB. Copyright © 2016 Pandey et al. 2016 Pandey et al. This content is distributed under the terms of the Creative Commons Attribution 4.0 International license.

10.1128/mSphere.00132-16.7 Table S3  Summary and annotation of all polymorphic loci detected in MDV-1 consensus genomes. Download Table S3, XLSX file, 0.1 MB. Copyright © 2016 Pandey et al. 2016 Pandey et al. This content is distributed under the terms of the Creative Commons Attribution 4.0 International license.

**TABLE 2:** Pairwise DNA identity and variant proteins between pairs of consensus genomes

Comparison	% DNA identity	Total no. of bp different	No. of intergenic:		No. of genic^a^:		
			Indels (events)	SNPs	Indels (events)	Synonymous SNPs	Nonsynonymous SNPs
**Different farms, dust vs dust**							
Farm B-dust vs Farm A-dust 1	99.73	353	143 (22)	140	66 (1) in DNA-pol	1 in helicase-primase	3 (1 each in vLIP, LORF2, and UL43)
Farm B-dust vs Farm A-dust 2	99.87	195	49 (14)	76	66 (1) in DNA-pol	1 in helicase-primase	3 (1 each in vLIP, LORF2, and UL43)
**Same farm, same time, dust vs host**							
Farm B-dust vs Farm B-feather 1	99.64	552	476 (11)	6	66 (1) in DNA-pol	1 in helicase-primase	3 (1 each in vLIP, LORF2, and UL43)
Farm B-dust vs Farm B-feather 2	99.52	687	572 (19)	45	66 (1) in DNA-pol	1 in helicase-primase	3 (1 each in vLIP, LORF2, and UL43)
**Same farm separated in time and space**							
Farm A-dust 1 vs Farm A-dust 2	99.76	338	170 (20)	168	0	0	0
**Same farm, same time, 1 host vs another**							
Farm B-feather 1 vs Farm B-feather 2	99.38	973	972 (9)	1	0	0	0

a DNA-pol, DNA polymerase processivity subunit protein UL42 (MDV055); helicase-primase, helicase-primase subunit UL8 (MDV020); vLIP, lipase homolog (MDV010); LORF2, immune evasion protein (MDV012); UL43, probable membrane protein (MDV056).

### DNA and amino acid variations between five new field genomes of MDV-1.

We began our assessment of genetic diversity by determining the extent of DNA and amino acid variations between the five different consensus genomes. We found that the five genomes are highly similar to one another at the DNA level, with the percentage of homology ranging from 99.4% to 99.9% in pairwise comparisons (Fig. 2C; Table 2). These comparisons used a trimmed-genome format (Fig. 2B) in which the terminal repeat regions had been removed, so that these sequences were not overrepresented in the analyses. The level of identity between samples is akin to that observed in closely related isolates of herpes simplex virus 1 (HSV-1) (39). Observed nucleotide differences were categorized as genic or intergenic and further subdivided based on whether the differences were insertions or deletions (indels) or single-nucleotide polymorphism (SNPs) (Table 2). The number of nucleotide differences was higher in intergenic regions than in genic regions for all genomes. For the indel differences, we also calculated the minimum number of events that could have led to the observed differences, to provide context on the relative frequency of these loci in each genome. We anticipate that these variations include silent mutations, as well as potentially advantageous or deleterious coding differences.

To understand the effect(s) of these nucleotide variations on protein coding and function, we next compared the amino acid sequences of all open reading frames (ORFs) for the five isolates. The consensus protein coding sequences of all five isolates were nearly identical, with just a few differences (Table 2). In comparison to the other four samples, Farm B-dust harbored amino acid substitutions in four proteins. A single nonsynonymous mutation was seen in each of the following: the virulence-associated lipase homolog vLIP (MDV010; Farm B-dust, S501A) (40), the major histocompatibility complex (MHC) class I immune evasion protein LORF2 (MDV012; Farm B-dust, L311W) (41), and the probable membrane protein UL43 (MDV056; Farm B-dust, S74L). A single synonymous mutation was observed in the DNA helicase-primase protein UL8 (MDV020; Farm B-dust, L253L). Finally, a 22-amino-acid (aa) insertion unique to Farm B-dust was observed in the DNA polymerase processivity subunit protein UL42 (MDV055; Farm B-dust, insertion at aa 277). We did not observe any coding differences between temporally separated dust isolates from Farm A or between feather isolates from different hosts in Farm B, although both of these comparisons (Table 2, bottom) revealed hundreds of noncoding differences.

### Detection of polymorphic bases within each genome.

Comparison of viral genomes found in different sites provides a macrolevel assessment of viral diversity. We next investigated the presence of polymorphic viral populations within each consensus genome to reveal how much diversity might exist within a field site (as reflected in dust-derived genomes) or within a single host (as reflected in feather genomes).

For each consensus genome, we used polymorphism detection analysis to examine the depth and content of the sequence reads at every nucleotide position in each genome (see Materials and Methods for details). Rather than detecting differences between isolates, as in Table 2, this approach revealed polymorphic sites within the viral population that contributed to each consensus genome. We detected 2 to 58 polymorphic sites within each consensus genome (Fig. 3) (see Materials and Methods for details). The feather genomes had a lower number of polymorphisms than the dust genomes, which may be due to low within-follicle diversity or the relatively low sequence coverage. Indels were not included in this polymorphism analysis but clearly contributed to between-sample variation (Table 2), suggesting that this may be an underestimate of the overall amount of within-sample variation. Viral polymorphisms were distributed across the entire length of the genome (Fig. 3), with the majority concentrated in the repeat regions. Application of a more stringent set of parameters (see Materials and Methods for details) yielded a similar distribution of polymorphisms, albeit with no polymorphisms detected in feather samples due to their lower depth of coverage (see Fig. S3 in the supplemental material). These data reveal that polymorphic alleles are present in field isolates, including in viral genomes collected from single sites of shedding in infected animals.

To address the potential effect(s) of these polymorphisms on MDV biology, we divided the observed polymorphisms into categories of synonymous, nonsynonymous, genic untranslated, or intergenic (see Table S3 in the supplemental material). The majority of all polymorphisms were located in intergenic regions (see Table S3). We next investigated whether evidence of selection could be detected from the distribution of polymorphisms in our samples. One way to assess this is to determine whether the relative frequencies of synonymous, nonsynonymous, genic untranslated, and intergenic polymorphisms can be explained by random chance. If the observed frequencies differ from those expected from a random distribution, it would suggest genetic selection. After calculating the expected distribution in each sample (as described in Materials and Methods), we determined that the distribution of variants differed from that expected by chance in each of our dust samples (Fig. 4, Farm A-dust 1, χ^2^ = 68.16, df =3, *P* < 0.001; Farm A-dust 2, χ^2^ = 128.57, df =3, *P* < 0.001; Farm B-dust 1, χ^2^ = 63.42, df =3, *P* < 0.001). In addition, we found in pairwise tests that the number of observed intergenic polymorphisms was significantly higher than the observed values for other categories (see Table S4 in the supplemental material). This suggests that the mutations that occurred in the intergenic regions were better tolerated and more likely to be maintained in the genome—i.e., that purifying selection was acting on coding regions.

10.1128/mSphere.00132-16.8 Table S4  Chi-square test values from pairwise comparisons of different categories of polymorphisms. Download Table S4, PDF file, 0.1 MB. Copyright © 2016 Pandey et al. 2016 Pandey et al. This content is distributed under the terms of the Creative Commons Attribution 4.0 International license.

### Tracking shifts in polymorphic loci over time.

**FIG 5:**
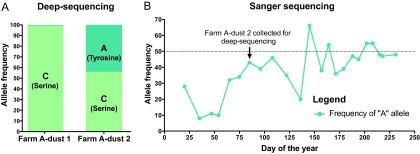
A new polymorphic locus in ICP4 and its shifting allele frequency over time. (A) HTS data revealed a new polymorphic locus in ICP4 (MDV084) at nucleotide position 5495. In the spatially and temporally separated dust samples from Farm A (see Fig. 1A and Materials and Methods for details), we observed different prevalences of C (encoding serine) and A (encoding tyrosine) alleles. (B) Using targeted Sanger sequencing of this locus, time-separated dust samples spanning 9 months were Sanger sequenced to track polymorphism frequency at this locus over time. The major and minor allele frequencies at this locus varied widely across time, and the major allele switched from C to A more than twice during this time.

In addition to observing polymorphic SNPs in each sample at a single moment in time, we explored whether any shifts in polymorphic allele frequency were detected in the two sequential dust samples from Farm A. We found one locus in the ICP4 (MDV084/MDV100) gene (nucleotide position 5495) that was polymorphic in the Farm A-dust 2 sample, with nearly equal proportions of sequence reads supporting the major allele (C) and the minor allele (A) (Fig. 5A). In contrast, this locus had been 99% A and only 1% C in Farm A-dust 1 (collected 11 months earlier in another house on the same farm), such that it was not counted as polymorphic in that sample by our parameters (see Materials and Methods for details). At this polymorphic locus, the nucleotide C encodes a serine, while nucleotide A encodes a tyrosine. The encoded amino acid lies in the C-terminal domain of ICP4 (aa 1832). ICP4 is an important immediate-early protein in all herpesviruses, where it serves as a major regulator of viral transcription (42–44). The role of ICP4 in MDV pathogenesis is also considered crucial because of its proximity to the latency-associated transcripts (LATs) and recently described miRNAs (44–46). In a previous study of MDV-1 attenuation through serial passage *in vitro*, mutations in ICP4 appeared to coincide with attenuation (31).

Given the very different allele frequencies at this ICP4 locus between two houses on the same farm 11 months apart, we examined dust samples from one of the houses over 9 months with targeted Sanger sequencing of this SNP (Fig. 5B). We found that this locus was highly polymorphic in time-separated dust samples. The A (tyrosine) allele rose to almost 50% frequency in the 9-month period. In four of the dust samples, the A (tyrosine) allele was dominant over the C (serine) allele. This reversible fluctuation in allele frequencies over a short period of time is unprecedented for alphaherpesviruses so far as we know. However, recent studies on human cytomegalovirus (HCMV) have shown that selection can cause viral populations to evolve in short periods of time (34, 35). While this is only one example of a polymorphic locus that shifts in frequency over time, similar approaches could be used at any of the hundreds of polymorphic loci detected here (see Table S3 in the supplemental material).

### Comparison of field isolates of MDV-1 to previously sequenced isolates.

**FIG 6:**
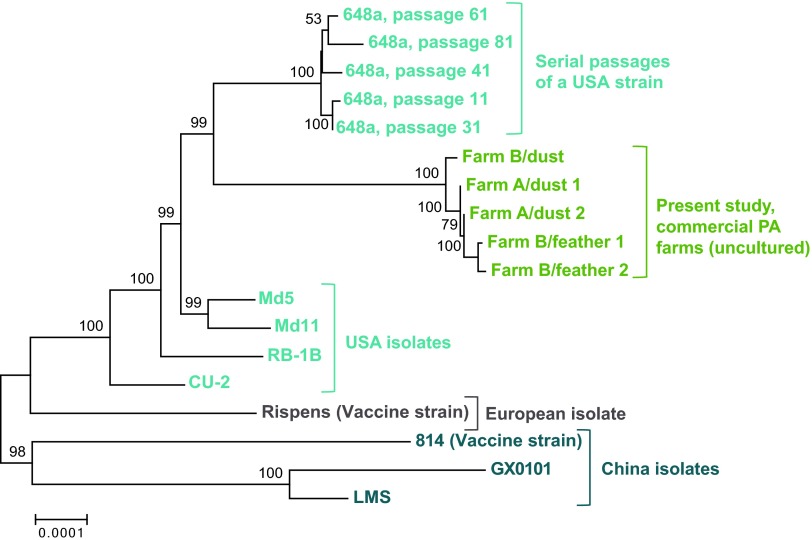
Dendrogram of genetic distances among all sequenced MDV-1 genomes. Using a multiple-genome alignment of all available complete MDV-1 genomes, we calculated the evolutionary distances between genomes using the Jukes-Cantor model. A dendrogram was then created using the neighbor-joining method in MEGA with 1,000 bootstraps. The five new field-sampled MDV-1 genomes (green) formed a separate group between the two clusters of United States isolates (blue). The European vaccine strain (Rispens) formed a separate clade, as did the three Chinese MDV-1 genomes (aqua). GenBank accession numbers for all strains are as follows: new genomes, listed in Table 1; passage 11 648a, JQ806361; passage 31 648a, JQ806362; passage 61 648a, JQ809692; passage 41 648a, JQ809691; passage 81 648a, JQ820250; CU-2, EU499381; RB-1B, EF523390; MD11, 170950; Md5, AF243438; Rispens (CVI988), DQ530348; 814, JF742597; GX0101, JX844666; and LMS, JQ314003.

To compare these new field-based MDV genomes to previously sequenced isolates of MDV, we created a multiple sequence alignment of all available MDV-1 genomes (14, 15, 17–21, 47, 48). The multiple sequence alignment was used to generate a dendrogram depicting genetic relatedness (see Materials and Methods). We observed that the five new isolates form a separate group compared to all previously sequenced isolates (Fig. 6). This may result from geographic differences as previously seen for HSV-1 and VZV (27, 49–52), from temporal differences in the time of sample isolations, or from the lack of cell culture adaptation in these new genomes.

We also noted a distinctive mutation in the genes encoding glycoprotein L (gL; also known as UL1 or MDV013). All of the field isolates had a 12-nucleotide deletion in gL that has been described previously in strains from the eastern United States. This deletion is found predominantly in very virulent or hypervirulent strains (vv and vv+ in the MDV-1 pathotyping nomenclature [1]) (53–56). This deletion falls in the putative cleavage site of gL, which is necessary for its posttranslational modification in the endoplasmic reticulum (54). Glycoprotein L forms a complex with another glycoprotein, gH. The gH/gL dimer is conserved across the *Herpesviridae* family and has been associated with virus entry (57, 58).

These field-isolated genomes also contain a number of previously characterized variations in the oncogenesis-associated Marek’s EcoRI-Q-encoded protein (Meq; also known as MDV005, MDV076, and RLORF7). We observed three substitutions in the C-terminal (transactivation) domain of Meq (P153Q, P176A, and P217A) (59). The first two of these variations have been previously associated with MDV-1 strains of very virulent and hypervirulent pathotypes (vv and vv+) (53, 60, 61), while the third mutation has been shown to enhance transactivation (62). In contrast, the field isolates lacked the 59-aa insertion in the Meq proline repeats that is often associated with attenuation, as seen in the vaccine strain CVI988 and the mildly virulent strain CU-2 (47, 48, 63, 64). We also observed a C119R substitution in all five field-derived genomes, which is absent from attenuated and mildly virulent isolates. This C119R mutation falls in the LXCXE motif of Meq, which normally binds to the tumor suppressor protein Rb to regulate cell cycle progression (53, 65). Although comprehensive *in vitro* and *in vivo* studies will be required to fully understand the biological implications of these variations, sequence comparisons of both gL and mEq from dust and feather genomes suggest that these closely resemble highly virulent (vv and vv+) variants of MDV-1 (47, 48). This is corroborated by the dendrogram (Fig. 6), where the dust- and feather-derived genomes cluster closely with 648a, which is a highly virulent (vv+) MDV isolate.

### Assessment of taxonomic diversity in dust and feathers.

As noted in Table 1, only a fraction of the reads obtained from each sequencing library were specific to MDV-1. We analyzed the remaining sequences to gain insight into the taxonomic diversity found in poultry dust and feathers. Since our enrichment for viral capsids removed most host and environmental contaminants, the taxa observed here represent only a fraction of the material present initially. However, this provides useful insight into the overall complexity of each sample type. The results of the classification for the Farm B-dust, Farm B-feather 1, and Farm B-feather 2 samples are shown in Fig. S4 in the supplemental material. We divided the sequence reads by the different kingdoms they represent. Complete lists of taxonomic diversity for all samples to the family level are listed in Table S5 in the supplemental material. As expected, the taxonomic diversity of dust is greater than that of feather samples. The majority of sequences in the dust samples mapped to the chicken genome, and only about 2 to 5% were MDV specific (see also Table 1, percentage of MDV-specific reads). We found that single feathers were a better source of MDV DNA, due to their reduced level of taxonomic diversity and higher percentage of MDV-specific reads (Table 1; see Fig. S4).

10.1128/mSphere.00132-16.4 Figure S4  Taxonomic diversity in dust and chicken feathers from Farm B. We used an iterative BLASTN workflow to generate taxonomic profiles for all samples from Farm B (see Materials and Methods for details). Major categories are shown here, with a full list of taxa (to the family level) given in **Table S5**. Farm B-feather 1 and Farm B-feather 2 show less overall diversity, as would be expected from direct host sampling versus the environmental mixture of the dust samples. Since the viral DNA enrichment procedures remove variable amounts of host and environmental contaminants, the proportion of taxa present is representative but not fully descriptive of those present initially. The asterisk indicates sequences that were unclassified or at low prevalence. Download Figure S4, PDF file, 0.9 MB. Copyright © 2016 Pandey et al. 2016 Pandey et al. This content is distributed under the terms of the Creative Commons Attribution 4.0 International license.

10.1128/mSphere.00132-16.9 Table S5  Summary of taxonomic classification to the family level for all samples. Download Table S5, XLSX file, 0.04 MB. Copyright © 2016 Pandey et al. 2016 Pandey et al. This content is distributed under the terms of the Creative Commons Attribution 4.0 International license.

## DISCUSSION

This study presents the first description of MDV-1 genomes sequenced directly from a field setting. This work builds on recent efforts to sequence VZV and HCMV genomes directly from human clinical samples, but importantly the approaches presented here do not employ either the oligonucleotide enrichment used for VZV or the PCR amplicon strategy used for HCMV (10, 33–35, 66). This makes our technique widely accessible and reduces potential methodological bias. It is also more rapid to implement and is applicable to the isolation of unknown large DNA viruses, since it does not rely on sequence-specific enrichment strategies. These five genomes were interrogated at the level of comparing consensus genomes—between-host variation—as well as within each consensus genome—within-host variation. By following up with targeted PCR and Sanger sequencing, we demonstrate that HTS can rapidly empower molecular epidemiological field surveillance of loci undergoing genetic shifts.

Although a limited number of nonsynonymous differences were detected between the field samples compared here, it is striking that several of these (vLIP, LORF2, and UL42) have been previously demonstrated to have roles in virulence and immune evasion. The N-glycosylated protein viral lipase (vLIP; MDV010) gene encodes a 120-kDa protein that is required for lytic virus replication in chickens (3, 40). The vLIP gene of MDV-1 is homologous to those of other viruses in the *Mardivirus* genus as well as to avian adenoviruses (67–69). The S501A mutation in the second exon of vLIP protein is not present in the conserved region that bears homology to other pancreatic lipases (40). The gene coding for the viral protein LORF2 (MDV012) is a viral immune evasion gene that suppresses MHC class I expression by inhibiting TAP transporter delivery of peptides to the endoplasmic reticulum (41). LORF2 is unique to the nonmammalian *Mardivirus* clade, but its function is analogous to that of the mammalian alphaherpesvirus product UL49.5 (41, 70, 71). Another study has shown that LORF2 is an essential phosphoprotein with a potential role as a nuclear/cytoplasmic shuttling protein (72). Interestingly, we also observed a 22-aa insertion in the DNA polymerase processivity subunit protein UL42 (MDV055). In herpesviruses, UL42 has been recognized as an integral part of the DNA polymerase complex, interacting directly with DNA and forming a heterodimer with the catalytic subunit of the polymerase (73–75). In HSV-1, the N-terminal two-thirds of UL42 has been shown to be sufficient for all known functions of UL42; the insertion in Farm B-dust falls at the edge of this N-terminal region (76). The nonsynonymous mutations and insertions detected here warrant further study to evaluate their impacts on protein function and viral fitness *in vivo*. The fact that any coding differences were observed in this small sampling of field-derived genomes suggests that the natural ecology of MDV-1 may include mutations and adaptations in protein function, in addition to genetic drift.

Drug resistance and vaccine failure have been attributed to the variation present in viral populations (10, 33, 77). Polymorphic populations allow viruses to adapt to diverse environments and withstand changing selective pressures, such as evading the host’s immune system, adapting to different tissue compartments, and facilitating transmission between hosts (10, 26, 33–35, 66, 77, 78). Polymorphisms that were not fully penetrant in the consensus genomes, but that may be fodder for future selection, include residues in genes associated with virulence and immune evasion, such as ICP4 (Fig. 5), Meq, pp38, vLIP, LORF2, and others (see Table S3 in the supplemental material). The nonsynonymous polymorphism that we observed in Meq is a low-frequency variant present in the C-terminal domain (I201L) (Farm B-dust; see Table S3). However, a comparison of 88 different Meq sequences from GenBank and unpublished field isolates (A. S. Bell, D. A. Kennedy, P. A. Dunn, and A. F. Read, unpublished data) did not reveal any examples where leucine was the dominant allele; all sequenced isolates to date have isoleucine at position 201.

Previous studies have examined the accumulation of polymorphic loci in MDV-1 genomes after serial passage *in vitro* (30, 31). Overall, we found a similar quantity of polymorphisms in field-derived genomes to that found in these prior studies, but we did not find any specific polymorphic loci that were identical between field-derived and *in vitro*-passaged genomes (30, 31). The ICP4 (MDV084/MDV100), LORF2 (MDV012), UL42, and MDV020 genes contain polymorphisms in both field and serially passaged isolates, albeit at different loci (30, 31). It is noteworthy that these coding variations are detected despite signs of clearance of polymorphisms from coding regions (Fig. 4), as indicated by the higher-than-expected ratios of intergenic to coding polymorphisms in these genomes. Together these findings suggest that MDV-1 exhibits genetic variation and undergoes rapid selection in the field, which may demonstrate the basis of its ability to overcome vaccine-induced host resistance to infection (3, 5, 79, 80).

For the viral transactivation protein ICP4, we explored the penetrance of a polymorphic locus (nucleotide position 5495) both in full-length genomes and also via targeted sequencing over time. Most of the work on this region in the MDV-1 genome has actually focused on the LATs that lie antisense to the ICP4 gene (44, 45). This polymorphic locus could thus impact either ICP4’s coding sequence (aa 1832 serine versus tyrosine) or the sequence of the LATs. This variation in ICP4 lies in the C-terminal domain, which in HSV-1 has been implicated in the DNA synthesis, late gene expression, and intranuclear localization functions of ICP4 (81, 82). This combination of deep-sequencing genomic approaches to detect new polymorphic loci and fast gene-specific surveillance to track changes in SNP frequency over a larger number of samples illustrates the power of high-quality full genome sequences from field samples to provide powerful new markers for field ecology.

Our comparison of new field-isolated MDV-1 genomes revealed a distinct genetic clustering of these genomes, separate from other previously sequenced MDV-1 genomes (Fig. 6). This pattern may results from geographic and temporal drift in these strains or from the wild, virulent nature of these strains versus the adaptation(s) to tissue culture in all prior MDV-1 genome sequences. The impact of geography on the genetic relatedness of herpesvirus genomes has been previously shown for related alphaherpesviruses such as VZV and HSV-1 (27, 49–52). Phenomena such as recombination can also have an impact on the clustering pattern of MDV isolates. It is worth noting that the genetic distance dendrogram constructed here included genomes from isolates that were collected over a 40-year span, which introduces the potential for temporal drift (14, 15, 17–21, 47, 48). Agricultural and farming practices have evolved significantly during this time, and we presume that pathogens have kept pace. To truly understand the global diversity of MDV, future studies will need to include the impacts of recombination and polymorphisms within samples, in addition to the overall consensus genome differences reflected by static genetic distance analyses.

Prior studies have shown that when MDV is passaged for multiple generations in cell culture, the virus accumulates a series of mutations, including several that affect virulence (30). The same is true for the betaherpesvirus HCMV (25). Extended passage *in vitro* forms the basis of vaccine attenuation strategies, as for the successful vaccine strain (vOka) of the alphaherpesvirus VZV (83). Cultured viruses can undergo bottlenecks during initial adaptation to cell culture, and they may accumulate variations and loss-of-function mutations by genetic drift or positive selection. The variations and mutations thus accumulated may have little relationship to virulence and the balance of variation and selection in the field. We thus anticipate that these field-isolated viral genomes more accurately reflect the genomes of wild MDV-1 strains that are circulating in the field. The ability to access and compare virus from virulent infections in the field will enable future analyses of vaccine-break viruses.

Our data and approaches provide powerful new tools to measure viral diversity in field settings and to track changes in large DNA virus populations over time in hosts and ecosystems. In the case of MDV-1, targeted surveillance based on an initial genomic survey could be used to track viral spread across a geographic area or between multiple end users associated with a single parent corporation (Fig. 1B). Similar approaches could be implemented for public or animal health programs—for instance, to guide management decisions on how to limit pathogen spread and contain airborne pathogens. The ability to sequence and compare large viral genomes directly from individual hosts and field sites will allow a new level of interrogation of host-virus fitness interactions, which form the basis of host resistance to infection (Fig. 1B). Finally, the analysis of viral genomes from single feather follicles, as from single VZV vesicles, enables our first insights into naturally occurring within-host variation during infection and transmission (Fig. 1B). Evidence from tissue compartmentalization studies in HCMV and VZV suggests that viral genomes differ in distinct body niches (10, 35, 66). These new technique will enable us to ask similar questions about MDV-1 and to begin exploring the relative fitness levels of viruses found in different tissue compartments.

## MATERIALS AND METHODS

### Collection of dust and feathers.

Samples were collected from two commercial-scale farms in central Pennsylvania, where each poultry building housed 25,000 to 30,000 individuals (Fig. 1A). The poultry on both farms were the same breed and strain of colored (“red”) commercial broiler chickens from the same hatchery and company. Dust samples were collected into 1.5-ml tubes from fan louvers. This location contains less moisture and contaminants than floor-collected samples and represents a mixture of airborne virus particles and feather dander. Sequential samples from Farm A (Table 1) were collected 11 months apart, from adjacent houses on the same farm (Fig. 1A). Samples from Farm B (Table 1) were collected from a single house at a single point in time. Feathers were collected just before hosts were transported from the farms for sale, to maximize the potential for infection and high viral titer. At the time of collection, the animals were 10 to 12 weeks old. Ten individuals were chosen randomly throughout the entirety of one house for feather collection. Two feathers from each animal were collected from the axillary track (breast feathers). The distal 0.5- to 1.0-cm proximal shaft or feather tip, which contains the feather pulp, was snipped into a sterile 1.5-ml microtube containing a single sterile 5-mm steel bead (Qiagen). On return to the laboratory, tubes were stored at −80°C until processing. One feather from each animal was tested for the presence and quantity of MDV-1 (see below for quantitative PCR [qPCR] details). The remaining feathers from the two animals with the highest apparent MDV-1 titer were used for a more thorough DNA extraction (see below for details) and next-generation sequencing. Animal procedures were approved by the Institutional Animal Care and Use Committee of the Pennsylvania State University (IACUC protocol no. 46599).

### Viral DNA isolation from dust.

MDV nucleocapsids were isolated from dust as indicated in Fig. S1A in the supplemental material. Dust collected from poultry houses was stored in 50-ml polypropylene Falcon tubes (Corning) at 4°C until required. Five hundred milligrams of dust was suspended in 6.5 ml of 1× phosphate-buffered saline (PBS). To distribute the dust particles into solution and help release cell-associated virus, the mixture was vortexed vigorously until homogenous and centrifuged at 2,000 × *g* for 10 min. This supernatant was further agitated on ice for 30 s using a Sonica ultrasonic processor Q125 (probe sonicator with 1/8-in. microtip) set to 20% amplitude. It was then vortexed before being centrifuged for a further 10 min at 2,000 × *g*. To enrich viral capsids away from the remaining contaminants, the supernatant (approximately 5 ml in volume) was subjected to a series of filtration steps. First, we used a Corning surfactant-free cellulose acetate (SFCA) filter (0.8-µm pore) that had been soaked overnight in fetal bovine serum (FBS) to remove particles at the level of eukaryotic cells and bacteria. To remove smaller contaminants, the flowthrough was then passed through a Millipore Express Plus membrane vacuum filter (0.22-µm pore), and the membrane was subsequently washed twice with 2.5 ml of PBS. To remove contaminant DNA, the final filtrate (approximately 10 ml in volume) was treated with DNase (Sigma) at a concentration of 0.1 mg/ml for 30 min at room temperature. In the absence of DNase treatment, we observed a higher yield of viral DNA, but with much lower purity (data not shown). The MDV nucleocapsids present in the DNase-treated solution were captured on a polyethersulfone (PES) membrane (VWR) filter (0.1-µm pore). This filter membrane trapped the viral nucleocapsids, which are between 0.1 and 0.2 µm (84). An increased MDV purity, but ultimately reduced total nanograms of DNA yield, may be achieved by washing this membrane once with 2.5 ml PBS (see Table S1 in the supplemental material). In the future, samples with a higher percentage of MDV DNA could be obtained by applying these wash steps to all components of the sample pool. The membrane was then carefully excised using a sterile needle and forceps and laid exit side downwards in a sterile 5-cm-diameter plastic petri dish, where it was folded twice lengthwise. The “rolled” membrane was then placed into a 2-ml microtube containing 1.8 ml of lysis solution (ATL buffer and proteinase K from the Qiagen DNeasy blood and tissue kit). Digestion was allowed to proceed at 56°C for 1 h on an incubating microplate shaker (VWR) set to 1,100 rpm. The membrane was then removed, held vertically over a tilted sterile 5-cm-diameter plastic petri dish, and washed with a small volume of the lysis solution (from the 2-ml microtube). This wash was subsequently returned to the 2-ml microtube, and the tube was placed again on the heated shaker, where it was allowed to incubate overnight. The following day, the DNA was isolated as per the manufacturer’s instructions using the DNeasy blood and tissue kit (Qiagen). DNA was eluted in 200 µl DNase-free water. Ten to 14 aliquots of 500 mg each were used to obtain sufficient DNA for each dust sample (see Table S1). Quantitative PCR was used to assess the copy number of viral genomes in the resulting DNA. The total yield and percentage of MDV-1 versus MDV-2 DNA are listed in Table S1.

### Isolation of viral DNA from feather follicles.

The protocol for extraction of MDV DNA from feather follicles was optimized for the smaller input material and an expectation of higher purity (see Fig. S1B in the supplemental material). Sequential size filters were not used to filter out contaminants from feather follicles, since these direct host samples have fewer impurities than the environmental samples of dust. However, the feather follicle cells were encased inside the keratinaceous shell of the feather tip, which required disruption to release the cells. Each tube containing a single feather tip and one sterile 5-mm-diameter steel bead was allowed to thaw, and then 200 µl of PBS was added, and the sample was bead beaten for 30 s at 30 Hz using a Tissuelyser (Qiagen) (see Fig. S1B). Vigorous bead beating achieved the desired destruction of the follicle tip. To dissociate the cells, 80 µl of 2.5 mg/ml trypsin (Sigma) and 720 µl of PBS were then added (final trypsin concentration, 0.8 mg/ml), and the solution was transferred to a new sterile 2-ml microtube and incubated for 2 h at 37°C on a heated microplate shaker (VWR) set to 700 rpm. To release cell-associated virus, the suspension was then sonicated on ice for 30 s using a Sonica ultrasonic processor Q125 (probe sonicator with 1/8-in. microtip) set to 50% amplitude. DNase I was added to a final concentration of 0.1 mg/ml and allowed to digest for 1 h at room temperature to remove nonencapsidated DNA. An equal volume of lysis solution (ATL buffer and proteinase K from the Qiagen DNeasy blood and tissue kit) was added, and the sample was incubated overnight at 56°C on an incubating microplate shaker (VWR) set to 1,100 rpm. The following day, the DNA was isolated as per the manufacturer’s instructions using the DNeasy blood and tissue kit (Qiagen). While the overall amount of DNA obtained from feather follicles was lower than that obtained from pooled dust samples (see Table S2 in the supplemental material), it was of higher purity and was sufficient to generate libraries for sequencing (Table 1, sample preparation).

### Measurement of total DNA and quantification of viral DNA.

The total amount of DNA present in the samples was quantified by fluorescence analysis using a Qubit fluorescence assay (Invitrogen) following the manufacturer’s recommended protocol. MDV genome copy numbers were determined using serotype-specific quantitative PCR (qPCR) primers and probes, targeting either the MDV-1 pp38 (MDV073; previously known as LORF14a) gene or MDV-2 (SB-1 strain) DNA polymerase (UL42, MDV055) gene. The MDV-1 assay was designed by Sue Baigent and used the forward primer Spp38for (5′ GAGCTAACCGGAGAGGGAGA 3′), reverse primer Spp38rev (5′ CGCATACCGACTTTCGTCAA 3′), and MDV-1 probe (FAM-CCCACTGTGACAGCC-BHQ1 [where FAM is 6-carboxyfluorescein and BHQ-1 is black hole quencher 1]) (S. Baigent, personal communication). The MDV-2 assay is that of Islam et al. (85), but with a shorter minor groove binder (MGB) probe (FAM-GTAATGCACCCGTGAC-MGB) in place of their BHQ-2 probe. Real-time quantitative PCRs were performed on an ABI Prism 7500 Fast system with an initial denaturation of 95°C for 20 s followed by 40 cycles of denaturation at 95°C for 3 s and annealing and extension at 60°C for 30 s. Both assays included 4 µl of DNA in a total PCR volume of 20 µl with 1× PerfeCTa qPCR FastMix (Quanta Biosciences), forward and reverse primers at 300 nM, and TaqMan BHQ (MDV-1) or MGB (MDV-2) probes (Sigma and Life Sciences, respectively) at 100 nM and 200 nM, respectively. In addition, each qPCR mixture incorporated 2 µl bovine serum albumin (BSA) (Sigma). Absolute quantification of genomes was based on a standard curve of serially diluted plasmids cloned from the respective target genes. The absolute quantification obtained was then converted to concentration. Once the concentrations of the total DNA, MDV-1 DNA, and MDV-2 DNA present in the sample were known, we calculated the percentages of MDV-1 and MDV-2 genomic DNA in the total DNA pool (see Tables S1 and S2 in the supplemental material).

### Illumina next-generation sequencing.

Sequencing libraries for each of the isolates were prepared using the Illumina TruSeq Nano DNA Sample prep kit, according to the manufacturer’s recommended protocol for sequencing of genomic DNA. The genomic DNA inputs used for each sample are listed in Table 1. The DNA fragment size selected for library construction was 550 bp. All of the samples were sequenced on an in-house Illumina MiSeq using version 3 sequencing chemistry to obtain paired-end sequences of 300 by 300 bp. Base calling and image analysis was performed with the MiSeq Control Software (MCS) version 2.3.0.

### Consensus genome assembly.

As our samples contained DNA from many more organisms than just MDV, we developed a computational workflow (see Fig. S2 in the supplemental material) to preprocess our data prior to assembly. A local BLAST database was created from every *Gallid herpesvirus* genome available in GenBank. All sequence reads for each sample were then compared to this database using BLASTN (86) with a loose E value of ≤10^−2^ in order to computationally enrich for sequences related to MDV. These “MDV-like” reads were then processed for downstream genome assembly. The use of bivalent vaccine made it possible for us to readily distinguish sequence reads that resulted from the shedding of virulent MDV-1 versus vaccine virus (MDV-2 or HVT) strains. The overall DNA identity of MDV-1 and MDV-2 is just 61% (87). In a comparison of strains MDV-1 Md5 (NC_002229), and MDV-2 SB-1 (HQ840738), we found no spans of identical DNA greater than 50 bp (data not shown). This allowed us to accurately distinguish these 300- by 300-bp MiSeq sequence reads as being derived from either MDV-1 or MDV-2.

MDV genomes were assembled using the viral genome assembly VirGA (39) workflow, which combines quality control preprocessing of reads, *de novo* assembly, genome linearization and annotation, and postassembly quality assessments. For the reference-guided portion of viral genome assembly in VirGA, the *Gallid herpesvirus 2* (MDV-1) strain MD5 was used (GenBank accession no. NC_002229.3). These new genomes were named according to recent recommendations, as outlined by Kuhn et al. (88). We use shortened forms of these names throughout the article (see Table 1 for short names). The full names for all five genomes are as follows: (i) MDV-1 *Gallus domesticus*-wt/Pennsylvania, United States/2015/Farm A-dust 1; (ii) MDV-1 *Gallus domesticus*-wt/Pennsylvania, United States/2015/Farm A-dust 2; (iii) MDV-1 *Gallus domesticus*-wt/Pennsylvania, United States/2015/Farm B-dust; (iv) MDV-1 *Gallus domesticus*-wt/Pennsylvania, United States/2015/Farm B-feather 1; and (v) MDV-1 *Gallus domesticus*-wt/Pennsylvania, United States/2015/Farm B-feather 2. GenBank accession numbers are listed below and in Table 1. Annotated copies of each genome, in a format compatible with genome and sequence browsers, are available at the Pennsylvania State University ScholarSphere data repository: https://scholarsphere.psu.edu/collections/1544bp14j.

### Between-sample consensus genome comparisons.

Clustalw2 (37) was used to construct pairwise global nucleotide alignments between whole-genome sequences and pairwise global amino acid alignments between open reading frames. These alignments were utilized by downstream custom python scripts to calculate percentage of identity, protein differences, and variation between samples.

The proline-rich region of UL36 (also known as VP1/2 or MDV049), which contains an extended array of tandem repeats, was removed from all five consensus genomes prior to comparison. The amount of polymorphism seen in this region of UL36 is driven by fluctuations in the length of these tandem repeats, as has been seen in prior studies with other alphaherpesviruses such as HSV, VZV, and pseudorabies virus (PRV) (32, 48–50, 99, 100). Since the length of extended arrays of perfect repeats cannot be precisely determined by *de novo* assembly (22, 23, 26, 27), we excluded this region from pairwise comparisons of genome-wide variation. Genome alignments with and without the UL36 region removed are archived at the ScholarSphere site: https://scholarsphere.psu.edu/collections/1544bp14j.

### Within-sample polymorphism detection within each consensus genome.

VarScan v2.2.11 (89) was used to detect variants present within each consensus genome. To aid in differentiating true variants from potential sequencing errors (90), two separate variant-calling analyses were explored (10). Our main polymorphism detection parameters (used in Fig. 3 and 4; see Tables S3 and S4 in the supplemental material) were as follows: minimum variant allele frequency, ≥0.02; base call quality, ≥20; read depth at the position, ≥10; and number of independent reads supporting the minor allele, ≥2. Directional strand bias of ≥90% was excluded; a minimum of two reads in opposing directions was required. For comparison and added stringency, we also explored a second set of parameters (used in Fig. S3 in the supplemental material): minimum variant allele frequency, ≥0.05; base call quality, ≥20; read depth at the position, ≥100; and number of independent reads supporting the minor allele, ≥5. Directional strand bias of ≥80% was excluded. The variants obtained from VarScan were then mapped back to the genome to understand their distribution and mutational impact using SnpEff and SnpSift (91, 92). Polymorphisms in the proline-rich region of UL36 were excluded, as noted above.

### Testing for signs of selection acting on polymorphic viral populations.

For each of our five consensus genomes, which each represent a viral population, we classified the polymorphisms detected into categories of synonymous, nonsynonymous, genic untranslated, or intergenic, based on where each polymorphism was positioned in the genome. For these analyses (Fig. 4), we were only able to include polymorphisms detected in the three dust genomes, since the total number of polymorphisms obtained from feather genomes was too low for chi-square analysis. First, we calculated the total possible number of single nucleotide mutations that could be categorized as synonymous, nonsynonymous, genic untranslated, or intergenic. To remove ambiguity when mutations in overlapping genes could be classified as either synonymous or nonsynonymous, genes with alternative splice variants or overlapping reading frames were excluded from these analyses. This removed 25 open reading frames (approximately 21% of the genome). These tallies of potential mutational events were used to calculate the expected fraction of mutations in each category. We preformed chi-square tests on each data set to assess whether the observed distribution of polymorphisms matched the expected distribution. We also performed a similar analysis in pairwise fashion (see Table S4 in the supplemental material), to assess whether the fraction of variants differed from what would be expected by random chance. Pairwise combinations included the following: synonymous versus nonsynonymous, synonymous versus intergenic, synonymous versus genic untranslated, nonsynonymous versus intergenic, nonsynonymous versus genic untranslated, and intergenic versus genic untranslated. Statistically significant outcomes would suggest that recent or historical selection differed between those categories of variants.

### Sanger sequencing of polymorphic locus in ICP4.

A potential locus of active selection within the ICP4 (MDV084/MDV100) gene was detected during deep sequencing of Farm B-dust. This locus was examined using Sanger sequencing. An approximately 400-bp region of the ICP4 gene was amplified using a Taq PCR core kit (Qiagen) and the following primers at 200 nM: forward primer ICP4selF (5′ AACACCTCTTGCCATGGTTC 3′) and reverse primer ICP4selR (5′ GGACCAATCATCCTCTCTGG 3′). Cycling conditions included an initial denaturation of 95°C for 2 min, followed by 40 cycles of denaturation at 95°C for 30 s, annealing at 55°C for 30 s and extension at 72°C for 1 min, with a terminal extension at 72°C for 10 min. The total reaction volume of 50 µl included 10 µl of DNA and 4 µl bovine serum albumin (BSA [final concentration, 0.8 mg/ml]). Amplification products were visualized on a 1.5% agarose gel; the target amplicon was excised and then purified using the EZNA gel extraction kit (Omega Bio-Tek). Sanger sequencing was performed by the Penn State Genomics Core Facility utilizing the same primers as used for DNA amplification. The relative peak height of each base call at the polymorphic position was analyzed using the ab1PeakReporter tool (93).

### Genetic distance and dendrogram.

Multiple sequence alignments of complete MDV-1 (*Gallid herpesvirus 2*) genomes from GenBank and those assembled by our lab were generated using MAFFT (94). The evolutionary distances were computed using the Jukes-Cantor method (95), and the evolutionary history was inferred using the neighbor-joining method (96) in MEGA6 (97), with 1,000 bootstrap replicates (98). Positions containing gaps and missing data were excluded. The 18-strain genome alignment is archived at ScholarSphere (https://scholarsphere.psu.edu/collections/1544bp14j).

### Taxonomic estimation of non-MDV sequences in dust and feathers.

All sequence reads from each sample were submitted to a quality control preprocessing method to remove sequencing primers, artifacts, and areas of low confidence (39). Sequence annotation was performed using a massively iterative all-versus-all BLASTN (E value, ≤10^−2^) approach using the all-nucleotide database from NCBI. Only a portion of the total sequence read pool could be identified with confidence using this method. We then used *de novo* assembly to extend the length of these unidentified sequences, therefore elongating them into contigs. These were iterated through BLASTN again, which revealed alignment to repetitive regions of the *Gallus domesticus* (chicken) genome. Since the viral DNA enrichment procedures include a level of stochasticity in removal of host and environmental contaminants, the proportion of taxa present is not a definitive outline of those present initially. The results of these classifications are shown in Fig. S4 and listed in Table S5 in the supplemental material.

### Accession number(s).

GenBank accession numbers are listed here and in Table 1: Farm A-dust 1, KU173116; Farm A-dust 2, KU173115; Farm B-dust, KU173119; Farm B-feather 1, KU173117; and Farm B-feather 2, KU173118. Additional files used in this article, such as multiple sequence alignments of these genomes, are archived and available at ScholarSphere (https://scholarsphere.psu.edu/collections/1544bp14j).
