# Supplementary material for: DNA from Dust: Comparative Genomics of Large DNA Viruses in Field Surveillance Samples
Source: mSphere. 2016 Oct 5;1(5):e00132-16. doi: 10.1128/mSphere.00132-16 (PMC5064450; doi:10.1128/mSphere.00132-16)
Supplement: Figure S1 [file sph005162146sf1.pdf]

Supplemental Figure S1. Procedures for enrichment and isolation of MDV DNA from dust or individual feather follicles.

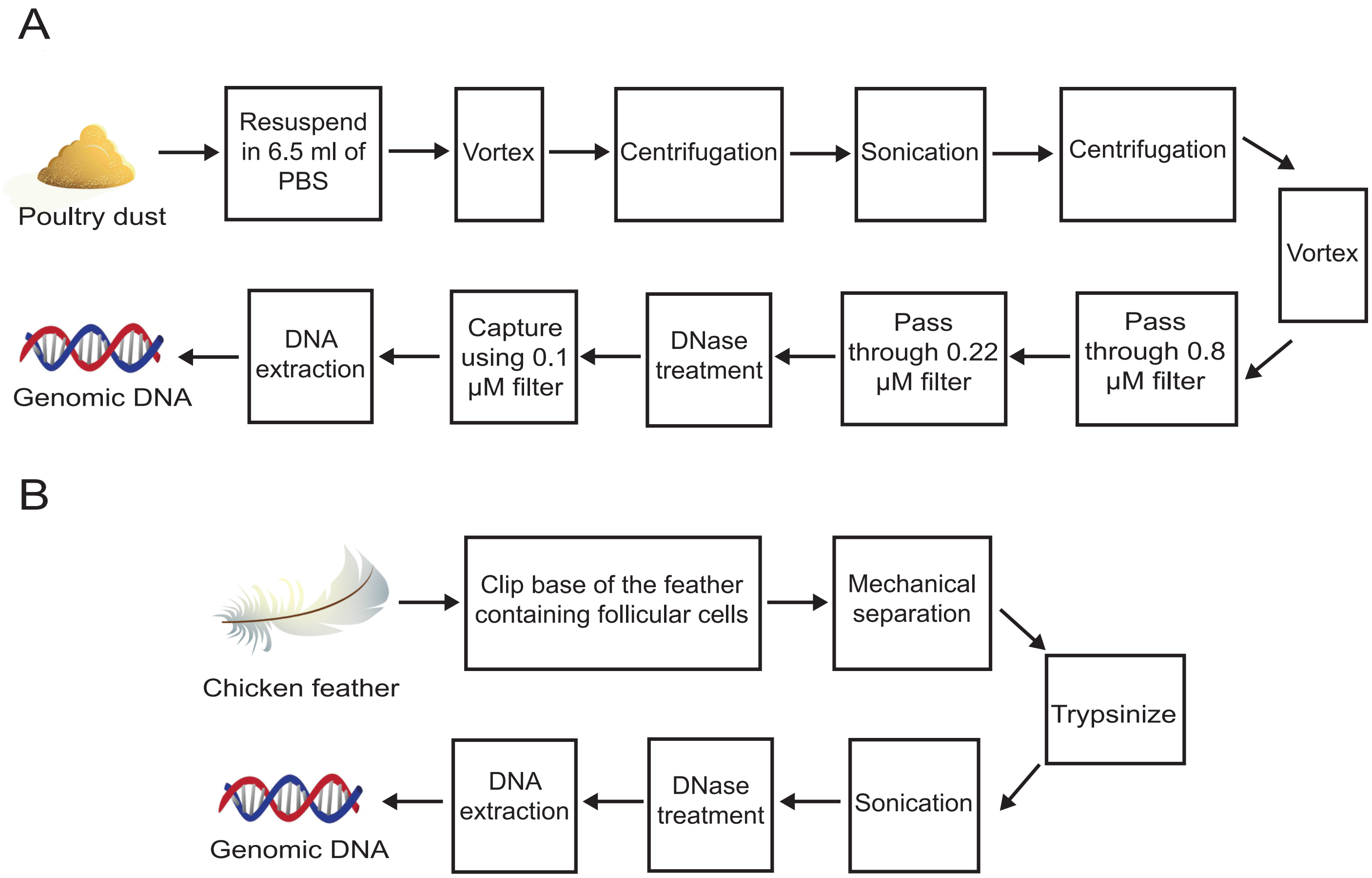

Pandey *et al.*, *mSphere* (2016).
DNA from dust: comparative genomics of large DNA viruses in field surveillance samples
