# Supplementary material for: DNA from Dust: Comparative Genomics of Large DNA Viruses in Field Surveillance Samples
Source: mSphere. 2016 Oct 5;1(5):e00132-16. doi: 10.1128/mSphere.00132-16 (PMC5064450; doi:10.1128/mSphere.00132-16)
Supplement: Table S1 [file sph005162146st5.pdf]

Supplemental Table S1: Yield and percent MDV-1+MDV-2 and total nanograms of DNA in each sample for Farm A-dust 1, Farm A-dust2, and Farm B-dust

Samples	Washes on 0.1 µm filter[a]	%MDV-1	%MDV-2	% MDV1 + MDV2	DNA (ng)
**Farm A-dust1**					
1	0	2.88	5.44	8.3	6.94
2	0	2.03	5.12	7.2	6.59
3	0	4.16	8.39	12.5	6.73
4	0	2.51	4.73	7.2	4.71
5	0	1.66	3.3	4.96	6.97
**6**	**1**	**9.13**	**13.99**	**23.12**	**2.69**
**7**	**1**	**9.29**	**15.7**	**24.99**	**2.16**
**8**	**1**	**5.86**	**10.91**	**16.77**	**3.36**
9	0	1.89	2.98	4.9	9.81
10	0	1.76	2.9	4.7	17.35
11	0	2.69	5.33	8.02	8.96
12	0	4.49	7.8	12.29	4.14
13	0	1.16	2.49	3.65	20
14	0	1.36	2.83	4.19	19.47
**Farm A-dust2**					
1	0	1.5	3.16	4.66	10.69
2	0	2.55	5.62	8.17	7.18
3	0	1.36	3.68	5.04	7.62
4	0	1.38	2.94	4.32	9.84
**5**	**1**	**2.71**	**6.19**	**8.9**	**4.11**
**6**	**1**	**3.08**	**5.87**	**8.95**	**4.37**
**7**	**1**	**2.68**	**4.91**	**7.59**	**5.88**
**8**	**1**	**3.49**	**6.24**	**9.73**	**4.88**
**9**	**1**	**4.09**	**7.94**	**12.03**	**2.66**
**10**	**1**	**6.42**	**10.52**	**16.94**	**3.15**
11	0	0.26	0.91	1.17	20.35
12	0	0.19	0.56	0.75	26.09
13	0	0.24	0.93	1.17	15.13
14	0	0.36	1.21	1.57	5.62
**Farm B-dust**					
1	0	0.84	6.68	7.52	14.1
2	0	0.46	5.2	5.66	26.64
3	0	0.65	4.85	5.5	19.43
4	0	0.75	5.91	6.66	16.84
5	0	0.23	3.67	3.9	25.9
6	0	0.53	4.65	5.18	23.5
**7**	**1**	**1.1**	**14.5**	**15.6**	**4.59**
**8**	**1**	**0.95**	**15.77**	**16.72**	**4.29**
**9**	**1**	**0.95**	**14.4**	**15.35**	**4.81**
**10**	**1**	**1.02**	**10.69**	**11.71**	**3.59**

[a]Samples that were washed before lysis (bold) yielded a higher percent MDV DNA, but less overall DNA
