# Supplementary material for: DNA from Dust: Comparative Genomics of Large DNA Viruses in Field Surveillance Samples
Source: mSphere. 2016 Oct 5;1(5):e00132-16. doi: 10.1128/mSphere.00132-16 (PMC5064450; doi:10.1128/mSphere.00132-16)
Supplement: Table S2 [file sph005162146st6.pdf]

Supplemental Table S2: Yield and percent MDV1+MDV2 and total nanograms of DNA in each sample for Farm B-feathers

Samples	% MDV-1	% MDV-2	% MDV-1 +MDV-2	DNA (ng)
Feather 1	40.59	0.12	40.72	11.97
Feather 2	5.68	0.02	5.70	27.36
