# Supplementary material for: DNA from Dust: Comparative Genomics of Large DNA Viruses in Field Surveillance Samples
Source: mSphere. 2016 Oct 5;1(5):e00132-16. doi: 10.1128/mSphere.00132-16 (PMC5064450; doi:10.1128/mSphere.00132-16)
Supplement: Table S4 [file sph005162146st8.pdf]

Supplemental Table S4: Chi-squared values from pairwise comparisons of different categories of polymorphisms.

Sample[a]	Intergenic vs. synonymous	Intergenic vs. non-synonymous	Intergenic vs. genic untranslated	Synonymous vs. non-synonymous	Synonymous vs. genic untranslated	Non-synonymous vs. genic untranslated
**Farm A-dust 1**	χ^2=16.6 (p = <0.001)	χ^2=55.47 (p = <0.001)	χ^2=3.74 (p = 0.053)	χ^2=0.03 (p=0.873)	χ^2=0.83 (p = 0.361)	χ^2=1.73 (p = 0.189)
**Farm A-dust 2**	χ^2=31.76 (p = <0.001)	χ^2=94.93 (p = <0.001)	χ^2=9.48 (p = 0.002)	χ^2=1.11 (p = 0.292)	χ^2=2.72 (p = 0.099)	χ^2=0.69 (p = 0.407)
**Farm B-dust**	χ^2=25.27 (p = <0.001)	χ^2=47.32 (p = <0.001)	χ^2=5.39 (p = 0.020)	χ^2=1.83 (p = 0.176)	χ^2=1.61 (p = 0.205)	χ^2=0.09 (p = 0.759)

[a]Degrees of freedom (d.f.) = 1 for all comparisons; p indicates p-value.
