# Supplementary figures and images for: DNA from Dust: Comparative Genomics of Large DNA Viruses in Field Surveillance Samples
Source: mSphere. 2016 Oct 5;1(5):e00132-16. doi: 10.1128/mSphere.00132-16 (PMC5064450; doi:10.1128/mSphere.00132-16)

**Supplemental Figure S4. Taxonomic diversity in dust and chicken feathers from Farm B.**

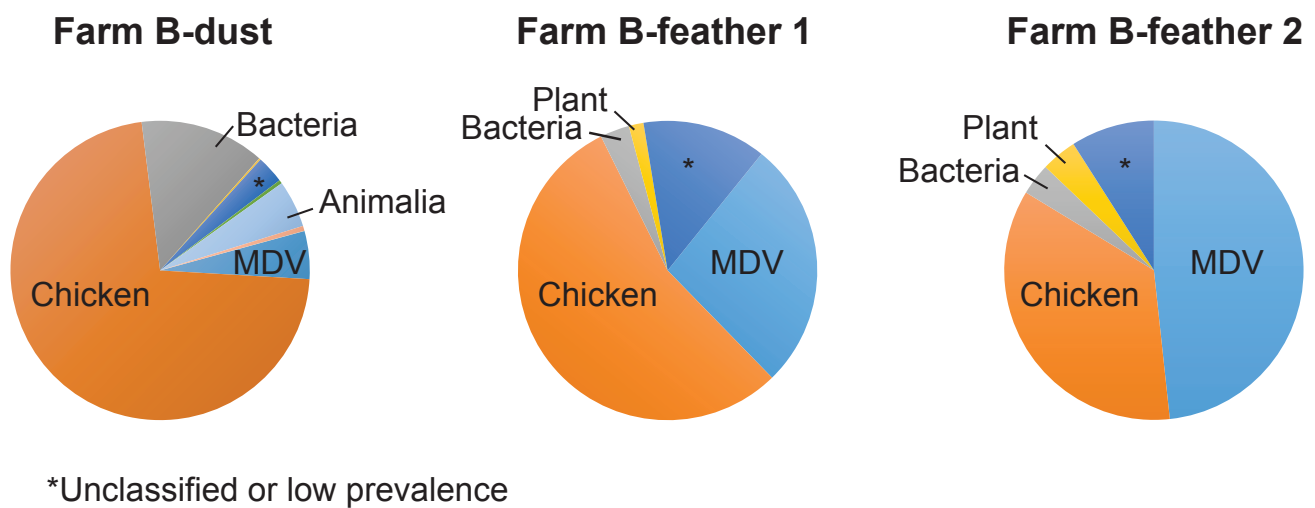

Supplement: Figure S4 [file sph005162146sf4.pdf]
